# Valve Abnormalities, Risk Factors for Heart Valve Disease and Valve Replacement Surgery in Spondyloarthritis. A Systematic Review of the Literature

**DOI:** 10.3389/fcvm.2021.719523

**Published:** 2021-09-24

**Authors:** Hye-Sang Park, Ana Laiz, Jesus Sanchez-Vega, Petra Díaz del Campo, Maria Auxiliadora Martín-Martínez, Mercedes Guerra-Rodríguez, Hector Corominas

**Affiliations:** ^1^Rheumatology Department, Hospital Dos de Maig, Barcelona, Spain; ^2^Rheumatology and Autoimmune Diseases Department, Hospital Universitari de la Santa Creu i Sant Pau, Barcelona, Spain; ^3^Department of Medicine, Universitat Autònoma de Barcelona (UAB), Barcelona, Spain; ^4^Cardiology Department, Hospital Universitari Bellvitge, Hospitalet de Llobregat, Spain; ^5^Department of Medicine, Universitat de Barcelona (UB), Barcelona, Spain; ^6^Research Unit, Spanish Society of Rheumatology, Madrid, Spain

**Keywords:** systematic (literature) review, HVD (heart valve disease), spondyloarthritis, echocardiography, cardiac image, epidemiology, psoriatic arthritis, valve (lesions, repair, replacement)

## Abstract

**Objective:** Evaluate the evidence on the abnormalities of the aortic root and heart valves, risk and prognostic factors for heart valve disease and valve replacement surgery in spondyloarthritis.

**Methods:** A systematic literature review was performed using Medline, EMBASE and Cochrane databases until July 2021. Prevalence, incidence, risk and prognostic factors for heart valve disease; dimension, morphology, and pathological abnormalities of the valves were analyzed. Patient characteristics (younger age, history of cardiac disease or longer disease duration) and period of realization were considered for the analysis. The SIGN Approach was used for rating the quality of the evidence of the studies.

**Results:** In total, 37 out of 555 studies were included. Overall, the level of evidence was low. The incidence of aortic insufficiency was 2.5–3.9‰. Hazard Ratio for aortic insufficiency was 1.8–2.0. Relative risk for aortic valve replacement surgery in ankylosing spondylitis patients was 1.22–1.46. Odds ratio for aortic insufficiency was 1.07 for age and 1.05 for disease duration. Mitral valve abnormalities described were mitral valve prolapse, calcification, and thickening. Aortic valve abnormalities described were calcification, thickening and an echocardiographic “subaortic bump.” Abnormalities of the aorta described were thickening of the wall and aortic root dilatation. The most common microscopic findings were scarring of the adventitia, lymphocytic infiltration, and intimal proliferation.

**Conclusions:** A higher prevalence and risk of aortic valve disease is observed in patients with ankylosing spondylitis. Studies were heterogeneous and analysis was not adjusted by potential confounders. Most studies did not define accurate outcomes and may have detected small effects as being statistically significant.

## Introduction

Spondyloarthropathies are a complex group of rheumatic diseases characterized by inflammation, erosion and new bone formations at peripheral and axial sites. The primary site of inflammation in spondyloarthropathies is thought to be the enthesis.

Another distinctive trait of spondyloarthritis is its overlap with other autoimmune organ diseases such as psoriasis, uveitis and inflammatory bowel disease. Textbooks and classical review articles also consider the heart as another organ frequently involved in spondyloarthritis and associated diseases (1, 2). Cardiac conduction disorders and valvular heart diseases are described as a specific cardiac manifestation of spondyloarthritis.

These statements are based on studies by Bergfeldt and other contemporary investigators. Bergfeldt was not the first investigator to describe cardiac manifestations in spondyloarthritis, but his work originated the concept of “HLA-B27 associated cardiac disease”. These studies were cross-sectional or case series describing HLA-B27 positivity or sacroiliitis in a group of patients with atrioventricular blocks, pacemakers and/or aortic regurgitations. Other frequently cited studies (3–8) were non adjusted case-control studies involving small sample sizes that compared the proportion of patients with valve disease in spondyloarthritis with control subjects. While the results may be interesting, the design and methods of the studies preclude the drawing of any associations between spondyloarthritis with atrioventricular blocks or aortic regurgitations. Furthermore, several recent studies have found no significant association between spondyloarthritis and heart valve diseases (9, 10). Therefore, we find that the evidence of heart valve disease in spondyloarthritis should be systematically reappraised.

Cardiac complications have been a research topic of growing interest over the last two decades. The frequent motto of research into the cardiac complications associated with rheumatic diseases is that chronic systemic inflammation may produce fibrosis in the myocardium and related structures (11). As publication in cardiac complications increased, a wide spectrum of cardiac complications has been described for systemic autoimmune diseases including heart valve diseases and conduction disorders (12, 13). Concurrently, cardiac complications spectrum in spondyloarthritis has also widened to ventricular dysfunction, myocarditis, pericarditis, heart failure or ischaemic heart disease (14). But are some cardiac complications more relevant in different rheumatic diseases?

Just as Bergfeldt and other authors marked a milestone in the investigation of spondyloarthritis, we similarly suspect that there may be a distinctive association between spondyloarthritis with heart valve disease. The aortic valve attachment site and peripheral entheses are known to have histological similarities (1). Two experimental studies observed that enthesis tissue resident T cells were also present in the aortic root and valve (15, 16) while being absent from the myocardium (15). Overexpression of interleukin-23 *in vivo* resulted in a dense infiltrate of T cells, macrophages and neutrophils in the attachment site of the aortic valve leading to the aortic wall as well as in the enthesis (15, 16). It can be hypothesized that inflammation of the valve attachment site may produce tissue degeneration that leads to aortic valve insufficiency.

This is the first systematic review of the literature to evaluate heart valve disease and valve replacement surgery in spondyloarthritis. The aim of this systematic review of the literature is to evaluate the existing evidence on the abnormalities of the aortic root and heart valves, as well as the risks and prognostic factors for heart valve disease and valve replacement surgery in spondyloarthritis. The objective of this study is to reappraise the presumption that heart valve disease is distinctive in patients with spondyloarthritis.

## Materials and Methods

A systematic review was conducted to identify all studies published up to December, 2020. This review was guided by the preferred reporting items for systematic review and meta-analysis (PRISMA) statement (Supplementary Material 1) (17).

### Search Strategy

A systematic search strategy was performed using the databases of Pubmed (Medline), EMBASE (Elsevier), and the Cochrane Library (Wiley) by a librarian (MG). The search strategy included MeSH terms and free text using different combinations (Supplementary Material 2). The search was limited to studies published until July 2021 in English, French, Korean, and Spanish. Additional references were manually retrieved by reviewing the references of the included studies. An update of the systematic research was performed before the submission of the manuscript.

### Inclusion Criteria

Studies were included if they had the characteristics described below:

**Population**: Patients with a diagnosis of SpA and fulfilling classification criteria of ASAS, ESSG, Amor, modified New York, ARA, Moll and Wright, or CASPAR criteria. Als, patients older than 18 years presenting grade II bilateral sacroiliitis or grade III or superior if unilateral sacroiliitis by X-Ray; and/or HLAB27 positivity; that also fulfilled two or more clinical characteristics (peripheral arthritis, enthesitis, dactylitis, uveitis, inflammatory back pain, psoriasis, inflammatory bowel disease, history of urethritis, or infective diarrhea) were included.
**Intervention**: valve insufficiency, regurgitation, dilatation, valvular bump, stenosis, annuloplasty, or valve replacement confirmed by cardiac imaging [transthoracic echocardiogram (TTE), transesophageal echocardiogram (TOE), or cardiac magnetic resonance (CMR)], post-mortem and/or surgical pathology or ICD codes.
**Outcomes**: Frequency measured by prevalence, prevalence ratio, incidence proportion and rate; Risk and prognostic factors measured by hazard ratio (HR), odds ratio (OR), risk ratio (RR), and risk difference (RD).
**Study design**: Meta-analysis, systematic review of the literature, case-control, cohort, cross sectional studies, and case series with more than five patients.

### Exclusion Criteria

Studies carried out in patients younger than 18 years, adolescents and pregnant women; studies not suited to the PICO framework in terms of the patient sample, intervention, comparison group(s), outcome(s) or study design; studies carried out in animals or populations based in ethnic minorities; and abstracts, posters, narrative reviews, letters, editorials, and any type of unpublished study.

### Article Selection

A total of 555 citations were peer reviewed by two rheumatologists (H.S.P and A.L) under the assessment of a senior methodologist (P.D and M.A.M). The reviewers independently performed two-stage screening (title/abstract and full-text screening), data extraction and risk of bias assessment. Some studies were not available mainly because of antiquity (Supplementary Material 3). They were previously consulted in two different national university library sources and the documentary collection of the Spanish Society of Rheumatology. The original authors were also contacted if possible. EndNote X8 software was used to manage the literature references.

### Data Extraction and Data Analysis

For data extraction, standardized forms from the Critical Appraisal Tools 3.0. Platform (http://www.lecturacritica.com/en/) was used. Risk of bias and quality of the evidence was rated according to the Scottish Intercollegiate Guidelines Network Approach (SIGN; http://www.sign.ac.uk/). Due to the small scale of the majority of the studies and heterogeneity in the design, a qualitative synthesis was carried out rather than a meta-analysis.

Some aspects were considered as critical to integrate the information. Specific characteristics of the population, such as younger age, history of cardiac disease or disease duration were contrasted for the results. A cardiologist specialized in cardiac imaging (J.S.V) was consulted for the interpretation of the data. Valve and aorta involvement was accepted only if confirmed by accurate techniques. Period of execution was also taken into account considering the evolution of imaging techniques throughout time.

## Results

**Figure 1:**
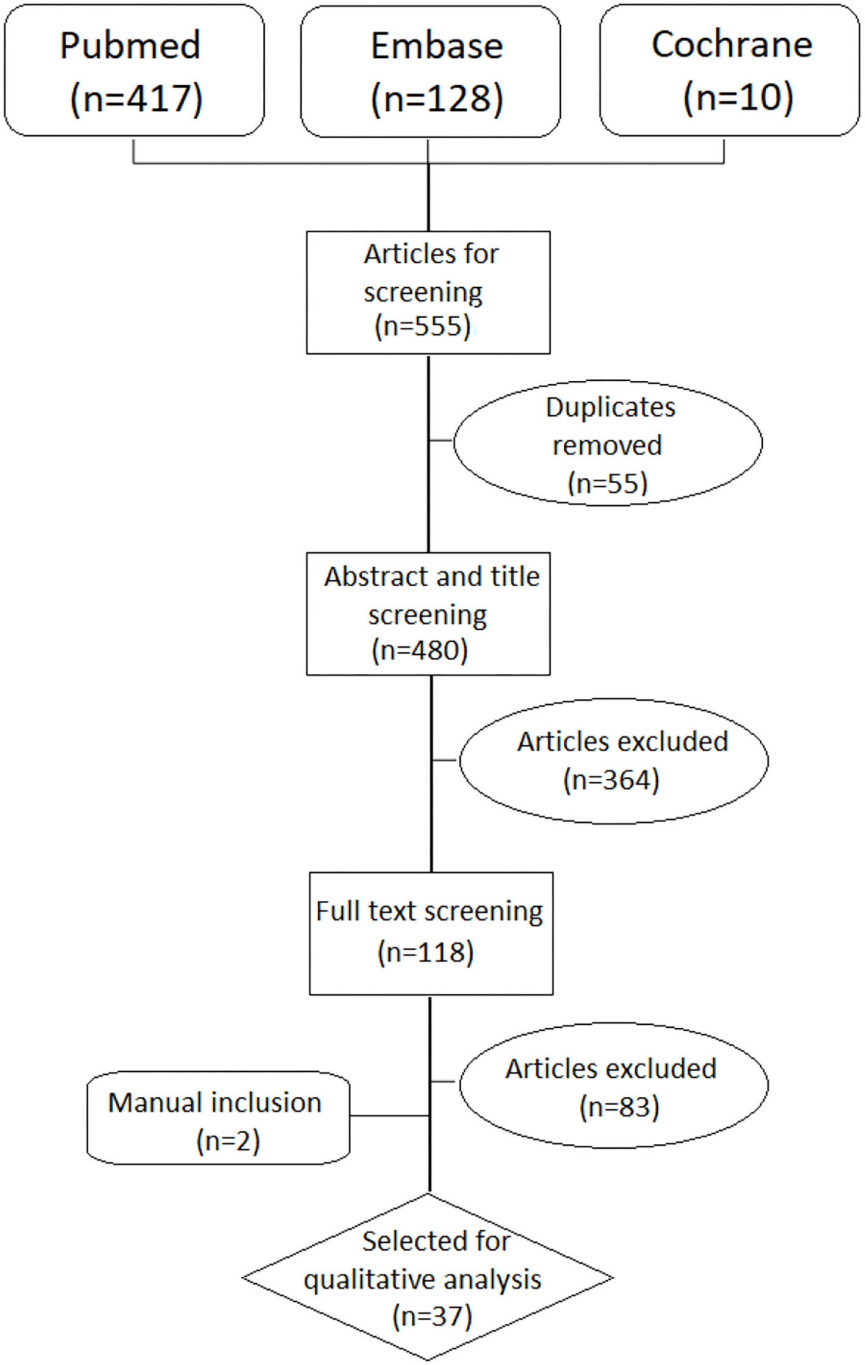
PRISMA flow chart.

Of the original 555 studies, we selected 118 studies for further reading based on title and abstract screening. After excluding 83 studies following full text reading, we included 37 studies in the analysis, as presented in the PRISMA flow-chart (Figure 1).

The reasons to exclude the remaining studies after reading the full text are provided in the Supplementary Material 4. Nineteen studies had a level of evidence 2- according to the SIGN grading system which implies high risk of confounding and non-causality. Seven studies were qualified as level 2+ with low risk for confounding and moderate probability for causality. Other eight studies were descriptive studies qualified as level 3. Details for each of the 35 studies are shown in Table 1.

**Table 1:** Characteristics of the studies included in the systematic literature review.

**References**	**Study type**	**Population**	**Sample size**	**Diagnosis**	**Outcomes**	**Quality (SIGN)**
Almasi et al. (18)	Case control	AS (excluding cardiac disease, cardiovascular risk factors and other comorbidities)	67 vs. 40	TTE Criteria of ASE	- Prevalence of mitral, aortic, tricuspid and pulmonar valve insufficiency or stenosis	2-
Alves et al. (19)	Cross sectional	AS	40	TTE Criteria of ASE & ESC guidelines	- Prevalence of Aortic insufficiency - Prevalence of mitral valve prolapse	3
Arnason et al. (20)	Case control	AS (excluding unstable angina or infarction <6 months)	29 vs. 13	TTE TOE Criteria of expert cardiologist	- Prevalence of aortic insufficiency - Morphologic findings of the valves and the aorta - Dimension of the aorta	2-
Bengtsson et al. (21)	Cohort	AS uSpA PsA	6,448 5,190 16,063	ICD	- Incidence of AoI and valve replacement surgery - HR for AoI	2+
Biesbroek et al. (22)	Cross sectional	AS (excluding if known cardiac disease)	16	TTE CMR Criteria aortic root dilatation if >4.1 cm	- Morphologic findings of the aorta - Dimension of the aorta	3
Brewerton et al. (23)	Case control	AS if age under 65 years (excluding cardiorespiratory disease)	30 vs. 30	TTE No criteria available	- Prevalence of valve disease - Morphologic findings of the valves and the aorta	2-
Brunner et al. (24)	Cross sectional	AS if more than 15 years of disease duration (excluding Pso, PsA or ReA)	100	TTE Criteria of valvular disease according to “The Echo Manual”	- Prevalence of aortic insufficiency	3
Bulkley and Roberts (25)	Case series	AS	8	Necropsy	- Pathology	3
Cantero Hinojosa et al. (26)	Case control	AS (excluding cardiovascular disease)	30 vs. 30	TTE Criteria of ASE guidelines	- Morphologic findings of the valves - Dimension of the aorta	2-
Crowley et al. (3)	Case control	AS (excluding cardiac disease or hypertension)	59 vs. 44	TTE Criteria of ASE guidelines	- Prevalence of valve disease	2-
de Almeida et al. (27)	Case control	SpA (excluding valve disease)	35 vs. 34	TTE Criteria of ASE guidelines	- Prevalence of aortic insufficiency - Morphologic findings of the valves and the aorta - Dimension of the aorta	2-
Eddarami et al. (28)	Cross sectional	AS	94	TTE	- Prevalence of aortic insufficiency - Morphologic findings of the valves and the aorta - Dimension of the aorta	3
Gonzalez Juanatey et al. (9)	Case control	PsA (excluding cardiovascular diseases and cardiovascular risk factors)	50 vs. 50	TTE Criteria of ASE guidelines	- Risk difference of valve disease - Dimension of the aorta	2+
Gunes et al. (10)	Case control	AS (excluding ischemic cardiac disease, cardiovascular risk factors and other comorbidities)	35 vs. 25	TTE Criteria of ASE guidelines	- Morphologic findings of the valves and the aorta - Dimension of the aorta	2-
Hannu et al. (29)	Cohort	ReA (excluding AS, PsA)	18	TTE Criteria of ASE guidelines	- Prevalence of valve disease - Morphologic findings of the aorta	2-
Kawasuji et al. (30)	Case series	AS	3	Necropsy	- Pathology - Surgical findings	3
Klingberg et al. (31)	Cohort	AS (excluding Pso, IBD, pregnancy or dementia)	187	TTE Criteria of ASE guidelines	- Measures of association with clinical variables, OR	2+
Klopf et al. (32)	Case control	AS	11 vs. 17	TTE Criteria not available	- Morphologic findings of the valves	2-
LaBresh et al. (33)	Case control	SpA	36 vs. 29	TTE Criteria not available	- Morphologic findings of the valves and the aorta - Dimension of the aorta	2-
Ljung et al. (34)	Cohort	AS	346	ICD	- Prevalence of valve disease - Measures of association with clinical variables, OR	2+
Midtbø et al. (35)	Case control	AS (excluding cardiovascular disease)	139 vs. 126	TTE Criteria according to EACVI and ASE guidelines	- Prevalence of aortic and mitral valve insufficiency	2+
Midtbø et al. (36)	Case control	AS (excluding cardiovascular disease)	106 vs. 106	TTE Criteria according to EACI & ASE guidelines	- Prevalence of aortic and mitral valve insufficiency - Aortic root diameter	2+
O'Neill et al. (37)	Case control	AS if more than 10 years of disease duration	24 vs. 24	TTE Criteria not available	- Prevalence of valve disease - Morphologic findings of the valves and the aorta	2-
Park et al. (38)	Case control	AS (excluding cardiac disease)	70 vs. 25	TTE TOE Criteria of ASE guidelines	- Morphologic findings of the valves - Dimension of the aorta and the valves	2-
Paulus et al. (39)	Case series	ReA	3	Necropsy Surgery	- Pathology - Surgical findings	3
Pines et al. (40)	Case control	PsA	25 vs. 32	TTE Criteria of expert cardiologist	- Prevalence of valve disease - Morphologic findings of the valves	2-
Qaiyumi et al. (41)	Case series	AS, ReA	7	Necropsy	- Pathology	3
Roldan et al. (7)	Case control	- AS if age under 60 years (excluding rheumatic fever, other SpA or iv drug abuse)	44 vs. 30	TTE TOE Criteria of clinically significant alteration numerically defined by investigators	- Measures of association, risk difference - Morphologic findings of the valves and the aorta - Dimension of the aorta and the valves	2-
Siao et al. (42)	Cohort	AS	3,780	ICD	- HR for valve disease - HR for valve replacement surgery	2+
Soroush et al. (43)	Case control	AS	50 vs. 40	TTE Criteria not available	- Prevalence - Measure of association, risk difference	2-
Sukenik et al. (44)	Case control	AS (excluding rheumatic fever or syphilis)	40 vs. 40	TTE Criteria not available	- Prevalence of valve disease	2-
Sun et al. (45)	Case control	AS (excluding cardiac diseas)	20 vs. 20	TTE Criteria not available	- Prevalence of valve disease	2-
Szabo et al. (4)	Cross sectional	AS during 1996–2006	8,616	ICD	- Prevalence - Measures of association: standardized prevalence ratio and risk of valvular heart disease by sex and age	3
Thomas et al. (46)	Case control	AS	23	TTE Criteria of expert cardiologist	- Prevalence of valve disease - Morphologic findings of the aorta	2-
Tucker et al. (47)	Case control	AS (Excluding known aortic valve disease)	35 vs. 20	TTE Criteria of ASE guidelines	- Morphologic findings of the valves and the aorta - Dimension of the aorta	2-
Ward (48)	Cohort	AS	19,254,030 (42,327 vs. 19,211,703)	ICD	- Prevalence of Aortic insufficiency and mitral insufficiency - Incidence valve replacement surgery - RR valve replacement surgery	2+
Yildirir et al. (8)	Case control	AS (excluding cardiac disease, cardiovascular risk factors and other rheumatic diseases)	88 vs. 31	TTE Criteria of ASE guidelines	- Prevalence of valve disease - Measures of association, risk difference - Morphologic findings of the valves and the aorta - Dimension of the aorta	2-

### Frequency, Risk, and Prognostic Factors for Heart Valve Disease

#### Prevalence

The prevalence of valve insufficiency and stenosis were described in patients with ankylosing spondylitis (AS), psoriatic arthritis (PsA), Reiter's Syndrome (ReS) and undifferentiated spondyloarthritis (uSpA). The majority of studies retrieved were small scaled with the exception of some nationwide health databases. Also, some studies were carried out in specific groups: patients without known cardiac disease, patients aged under 60, or with more than 10 years of disease duration. Period of execution and imaging technique were considered for the synthesis of the data.

In the general AS population the reported prevalence of aortic insufficiency was 3.3–18% (19, 23, 28, 31, 32, 43, 44, 46). The prevalence of aortic stenosis was 0–4% (31, 43), of mitral insufficiency was 2–74% (31, 43), of mitral stenosis was 0–2% (19, 32), of tricuspid insufficiency was 0–94% (31, 43), and of pulmonary insufficiency was 0–43% (31, 43). Only one study reported prevalence in relation to the degree of severity (31) which was 4.8% for moderate aortic insufficiency, 0.53% for severe aortic insufficiency, 1% for moderate mitral insufficiency, 3% for moderate tricuspid insufficiency, and 2% for moderate pulmonary insufficiency.

Three studies based on health insurance databases reported a prevalence of 1.1–3.9% which is lower than those based on small samples (4, 21, 34) probably due to the difference in the diagnostic method and severity of valve disease. One health insurance database reported the prevalence of aortic and mitral valve disease by age intervals but did not distinguish insufficiency from stenosis (48). The prevalence of aortic valve disease was 2.6% between 65 and 69 years, 6.7% between 70 and 74 years, 10.9% between 75 and 79 years and 17.1% in age 80 or older (48). The prevalence of mitral valve disease was 3.8% between 65 and 69 years, 9.8% between 70 and 74 years, 14.1% between 75 and 79 years and 19.2% in age 80 or older.

Four studies described the prevalence of valve disease in groups of young AS patients aged under 60 (4, 7, 18, 23, 28). They observed aortic insufficiency in 3.3–16%, mitral insufficiency in 0–32%, pulmonary insufficiency in 3% and tricuspid insufficiency in 2–77.6%. Aortic insufficiency was mild in 9.1%, moderate in 6.8–9% (7, 18). Mitral insufficiency was mild in 20.5% and moderate in 9–11.4% (7, 18). Mitral valve stenosis was observed in 1.5% (18).

Eight studies described the prevalence of valve disease in AS patients without known cardiac disease or symptoms (3, 8, 10, 20, 35, 36, 38, 45). They observed aortic insufficiency in 0–34.5% and mitral insufficiency in 0–49%. Moderate aortic insufficiency was observed in 2.3–10.3%.

In two studies based on AS patient groups with more than 10 years of disease duration, aortic insufficiency was observed in 8.3–10% (24, 37) of which moderate in 3% (24). Mitral insufficiency was observed in 29% of which 2% was moderate and 1% severe (24).

As for PsA the reported prevalence was wide ranged. A study based on a nationwide population registry detected 0.4% prevalence of aortic insufficiency. In a study of small samples the prevalence of mild aortic insufficiency was 10%, mild mitral insufficiency was 16% and mild tricuspid insufficiency was 10% (9). In another study no valve disease was observed at all (40).

In a small study of ReA population mild aortic insufficiency was seen in 5.6% and mild mitral insufficiency in 5.6% (29).

One study carried out in a mixed population of spondyloarthritis (AS, PsA, ReA) reported a prevalence of aortic insufficiency of 11.4%, which was mild in 8.5% and moderate in 2.9%. Mild mitral insufficiency was reported in 8.6% (27).

Standardized prevalence ratio for aortic valvular heart disease was 1.59 (95% IC 1.31–1.91) and for non-aortic valvular heart disease was 1.58 (95% IC 1.43–1.74%) (4).

Prevalence difference when compared to control groups showed contrasting results. Two studies carried out in an AS group younger than 60 years without known cardiac disease showed significantly higher prevalence for aortic insufficiency (7, 8) and mitral insufficiency (8) in AS groups.

However, six other studies based on PsA and AS groups without cardiac disease showed no significant difference in valve disease compared to control groups (9, 18, 35, 36, 38, 43).

#### Incidence

Two studies reported on the incidence of aortic insufficiency for AS, PsA and uSpA based on a nationwide population registry (21, 48). The incidence proportion of aortic insufficiency was 3.8‰ for AS, 2.5‰ for undifferentiated SpA and 3.9‰ for PsA (21). The incidence rate (1,000 person-years at risk) of aortic insufficiency was 0.7 (0.4–0.9) for AS, 0.5 (0.2–0.7) for uSpA, and 0.7 (0.5–0.8) for PsA (21).

#### Risk of Heart Valve Disease in Patients With SpA

When compared to a control population without AS, age and sex adjusted HR for aortic insufficiency was significantly increased in AS (HR 1.9, 95% IC 1.3–2.9), uSpA (HR 2.0, 95% IC 1.2–3.5), and PsA (HR 1.8, IC 1.4–2.4) (21). Similar results was observed in a nationwide population registry in patients with AS with a HR of 1.67 (95% CI 1.38–2.02, *p* < 0.001) when adjusted by confounders (28).

#### Risk or Prognostic Factors of SpA for Heart Valve Disease Manifestation

Three studies presented data about association of clinical SpA features with valve disease presentation (7, 31, 34). Age and disease duration showed association in all three studies. The OR for the duration of symptoms was 1.05 (31, 34) and the OR for age was 1.07 (34). Also male sex (OR 2.57), NSAID treatment (OR 0.37) (34), mSASSS (OR 1.02), history of anterior uveitis (OR 2.72) showed statistically significant association (19). No statistically important association was found for: disease activity, severity, diabetes mellitus or smoking habits (31).

### Frequency and Risk for Valve Surgery

#### Prevalence and Incidence for Valve Surgery

The prevalence of valve replacement surgery in AS was reported as 0.6–2.5% (21, 44). The prevalence of aortic valve surgery in PsA by a nationwide population registry was 0.4% (21).

The incidence of aortic valve replacement or repairing in AS patients was 1.25 per 1,000 person-years in age between 65 and 69, 1.83 per 1,000 person-years in age between 70 and 74, 2.61 per 1,000 person-years in age between 75 and 79 and 2.79 per 1,000 person-year in age 80 or older (48). The incidence of mitral valve surgery was 0.54 per 1,000 person-years in age between 65 and 69, 0.73 per 1,000 person-years in age between 70 and 74, 0.72 per 1,000 person-years in age between 75 and 79 and 0.55 per 1,000 person-year in age 80 or older (48). The incidence increased substantially over time in patients with AS but this increment also occurred in the controls so the risk remained constant over time.

#### Risk for Valve Surgery

RR of aortic valve repairing or replacement surgery in AS patients compared to the control population was significantly increased at all ages (48). In a nationwide population registry the HR for valve replacement surgery after developing valvular heart disease was higher in AS patients compared to those without AS with a HR of 5.09 (95% CI 1.55–16.68, *p* = 0.007) (28).

At ages between age 65–69 RR was 1.34 (95% CI 1.10–1.63), between age 70–74 was 1.22 (95% CI 1.05–1.43), between 75 and 59 was 1.25 (95% CI 1.10–1.43) and 1.46 (95% CI 1.32–1.63) in age 80 or older (48). RR of mitral valve surgery in AS patients compared to the control population was not statistically significant in any age group (48). None of the studies retrieved described SpA disease associated risk or prognostic factors for valve repairment surgery.

### Morphology and Pathology of the Aortic and Mitral Valves

#### Morphology

A total of 12 studies reported morphologic abnormalities by cardiac imaging in mitral and aortic valves. Type of imaging technique and period of realization were considered to synthesize the information.

Mitral valve abnormalities described were mitral valve prolapse (8, 19, 32, 37, 40), mitral valve calcification (23, 27) and thickening of the mitral valve (7, 8, 27, 28). Mitral valve prolapse had a prevalence between 4.17 and 56%. In studies published in the 80's the prevalence ranged from 10 to 36.4% in AS (19, 32) and 56% in PsA (40). The other two studies published after the 90;s showed much lower prevalence of 4.17–5.7% but were carried out in AS without known cardiac disease (8, 37). When compared to a control group the differences in prevalence were irrelevant (8, 37).

Mitral valve calcification was observed in two studies based on AS and SpA population without known valve disease with a prevalence of 6.67–8.57%.

Thickening of the mitral valve was observed in two studies carried out on patients without known valve disease with a prevalence of 2.22–22.86% (8, 27, 28) and on patients younger than 60 years (7) with a prevalence of 34%. The latter study was carried out by TOE which is the most suitable imaging modality for mitral valve anatomy. Mitral valve thickening was more prevalent in AS patients when compared to a control group (7). The mean mitral valve thickening was 0.12–0.36 cm (7, 38).

Aortic valve abnormalities described were calcification, thickening and an echocardiographic “subaortic bump.” It is a distinctive finding most described in spondyloarthritis that consists in a thickening of the aortomitral junction from the aortic cusps to the anterior mitral leaflet.

Calcification of the aortic valve was seen in 8.57% of SpA patients without known cardiac disease (27).

Thickening of the aortic valve in the AS and SpA population without known cardiac disease was 10–20% by TTE (27, 33) and 41–77% by TOE (7, 38). Two studies showed higher prevalence of thickening in AS patients when compared to control groups (7, 38). The mean aortic valve thickness was 0.18–0.32 cm (7, 38).

Subaortic bump was studied by five studies. The range of prevalence was broad. Two studies reported a prevalence of 25–77% when measured with TOE (7, 20). Other three studies described a lower prevalence of 4–31% measured by TTE (26, 33, 47). When compared to a control group, subaortic bump was more prevalent in AS patients (7, 20).

#### Pathology

Macroscopic findings were thickening of aortic valve cusps (25, 30, 39, 41). The most common microscopic findings were scarring of the adventitia, lymphocytic infiltration and intimal proliferation of valve cusps (25, 30, 39).

### Morphology, Dimension, and Pathology of the Aorta

#### Morphology

Morphologic abnormalities of the aorta observed were thickening of the aortic wall and aortic root dilatation. The thickening of the aortic wall was evaluated in two studies (7, 37). The thickening of the posterior aortic wall had a prevalence of 4.17% by TTE (37) and 61.4% by TOE (7), but only the latter provided the definition of thickening as >2.2 mm. Aortic root dilatation prevalence was between 1.11 and 26.1% by TTE, 25% by TOE and 0% by CMR (7, 8, 10, 22, 23, 28, 29, 46).

Some reported lower prevalence between 2.99 and 4.5% (8, 10, 23, 29) and others reported higher prevalence between 12.5 and 26.1% (7, 22, 46). This wideness of range in prevalence was not explained by differences in population, year of publication, cardiac imaging test or criteria used to diagnose dilatation. Disease duration showed correlation with aortic root dilatation (8) but disease severity and spine deformity showed no significant difference of aortic root dilatation (46).

#### Dimension

Fourteen studies reported measurement data on aortic root diameter, ascending aorta diameter and aortic wall thickness.

Mean aortic root diameter in AS patients was 3.2–3.4 cm (8, 20, 36) and in PsA patients was 3 cm (9). Other studies described dimension at different sites of the aortic root: at the annulus 1.6–3.1 cm (7, 26, 33, 38, 47), at the sinotubular junction 2.4–3.3 (7, 12) and at the sinuses of Valsalva 3.4–3.7 cm (8, 26, 31). Some measured in mid cusp and above the cusp without specifying the exact place of measurement even though most followed American Society of Echocardiography guidelines. In mid cusp the diameter was 1.9–3.3 cm (26, 33, 47) and above the cusp 2.0–3.4 cm (26, 33, 47).

Case control studies also reported diameter in control groups (7, 9, 20, 26, 33, 36, 38, 47). Only two studies concluded that the difference was statistically significant with a mean difference of 0.2 cm (7, 8). The diameter was similar for AS patients regarding positivity for HLA-B27, presence of inflammatory activity or severe sacroiliitis (26). One study found a trend for increasing age and larger aortic root dimension but no significant relation for the duration of disease symptoms (47).

Ascending aorta diameters were reported in 5 studies. Mean diameters ranged from 2.4 to 4.7cm (10, 22, 27, 28, 38) in AS patients. Similar diameters were reported in ReA 2.7 cm and in PsA 2.9 cm (27).

Aortic wall thickness at the anterior wall ranged from 0.24 to 0.25 cm (7, 20) and at the posterior wall from 0.27 to 0.38 cm (7, 20).

#### Pathology

Macroscopic findings of the aorta were dilatation of the aortic root and ascending aorta (25, 30, 39, 41). Most common microscopic findings were scarring of the adventitia, lymphocytic infiltration and intimal proliferation (25, 30).

## Discussion

Prevalence and incidence of valve heart disease had a wide range of variability due to the small number of samples, heterogeneity in sample and outcome definitions. Some studies found a higher prevalence of valve disease when compared to control populations with low grade of evidence (7, 8) while others did not (9, 18, 35, 36, 38, 43). Key aspects in valve degeneration are age, mechanism of valve disease and grade of severity but very few studies provided these information (49, 50).

Non-adjusted studies based on nationwide health databases showed higher HR for aortic insufficiency and slightly higher RR for valve replacement surgery compared to the general population (7, 8, 21, 48). According to some studies, age and disease duration showed association with valve disease with weak evidence for causality (7, 31, 34). These may be potential confounders but this possibility was not analyzed.

Morphologic abnormalities of the mitral and aortic valves were: calcification, thickening, subaortic bump and mitral valve prolapse (7, 20, 26, 33, 47). Subaortic bump, mitral and aortic valve thickening were more frequent in SpA patients when compared to control groups (7, 33, 38). Morphologic abnormalities of the aorta were aortic root dilatation and thickening of the posterior aortic wall. The prevalence of these findings was wide ranging. No difference was observed when sorted by population, year of publication or type of cardiac imaging used.

The diameter of the aortic root and ascending aorta were similar to control groups (7, 9, 20, 26, 33, 36, 38, 47). No significant difference was found when comparing mean aortic root diameter regarding different AS disease characteristics.

Several critical points are raised for the interpretation of the results. Very few studies were adjusted by potential confounders. Nationwide databases adjusted by sex and age but lacked clinical information to analyze potential confounders. Smaller studies lacked power or did not use adjusted models for design. Results may be spurious or may not have detected association. Another important point to consider is the absence of clinically relevant outcome definition of valve disease and abnormality. The grade of severity, mechanism of valve disease and relevant abnormalities considering age-related degeneration are very important information that most of the studies omitted. Some studies found statistically significant differences in clinically irrelevant results. Some other potentially interesting results are difficult to interpret due to imprecise description of the results. The evidence is weak due to the low quality of the majority of the studies.

This is the first systematic review of the literature regarding heart valve disease and aortic abnormalities in spondyloarthritis. A cardiac imaging specialist was consulted for strict interpretation of clinically relevant information. Some limitations are to be considered. Meta-analysis was not possible due to the heterogeneity in the design and clinical measures employed. The incomplete retrieval of the bibliography may affect some of the results but the majority of the missing studies were old and probably of lower evidence quality.

## Conclusions

The conclusion of our study is that further studies with larger sample sizes, multivariate analysis, and a better definition of outcomes are needed. Data to assess valve and aortic diseases in spondyloarthritis is weak and may be spurious because of possible confounders.

## Data Availability Statement

The original contributions presented in the study are included in the article/Supplementary Material, further inquiries can be directed to the corresponding author/s.

## Author Contributions

MG-R performed the systematic literature search. HSP and AL were involved in data screening, extraction, analysis, and drafted the manuscript. PD, HC, and MM-M contributed substantially to the study conceptions, design, and critical revision of the article. JS-V was consulted for interpretation of the extracted data as a referent cardiac imaging expert. All authors read and approved the final manuscript.

## Conflict of Interest

AL has received speaker fees/honoraria from Abbvie, Lilly, Novartis, Pfizer, and UCB. HC has received speaker fees/honoraria from BMS, Gebro, MSD, Lilly, Novartis, Pfizer, Roche, Sanofi, and UCB and has participated in consulting for Abbvie, Amgen, Biogen, Celgene, Gilead, Kern, Pfizer, and Sanofi. The remaining authors declare that the research was conducted in the absence of any commercial or financial relationships that could be construed as a potential conflict of interest.

## Publisher's Note

All claims expressed in this article are solely those of the authors and do not necessarily represent those of their affiliated organizations, or those of the publisher, the editors and the reviewers. Any product that may be evaluated in this article, or claim that may be made by its manufacturer, is not guaranteed or endorsed by the publisher.

## Abbreviations

AS: Ankylosing Spondylitis
ReA: Reactive arthritis
PsA: Psoriatic Arthritis
uSpA: undifferentiated spondyloarthritis
SpA: spondyloarthritis
TTE: Transthoracic echocardiography
TOE: Transesophageal echocardiography
ASE: American Society of Echocardiography
EACVI: European Association of Cardiovascular Imaging.
