# Supplementary material for: Valve Abnormalities, Risk Factors for Heart Valve Disease and Valve Replacement Surgery in Spondyloarthritis. A Systematic Review of the Literature
Source: Front Cardiovasc Med. 2021 Sep 24;8:719523. doi: 10.3389/fcvm.2021.719523 (PMC8498574; doi:10.3389/fcvm.2021.719523)
Supplement: Supplementary file 1 [file Data_Sheet_1.docx]

Supplementary Material

**Figure A. PRISMA flow chart**

**
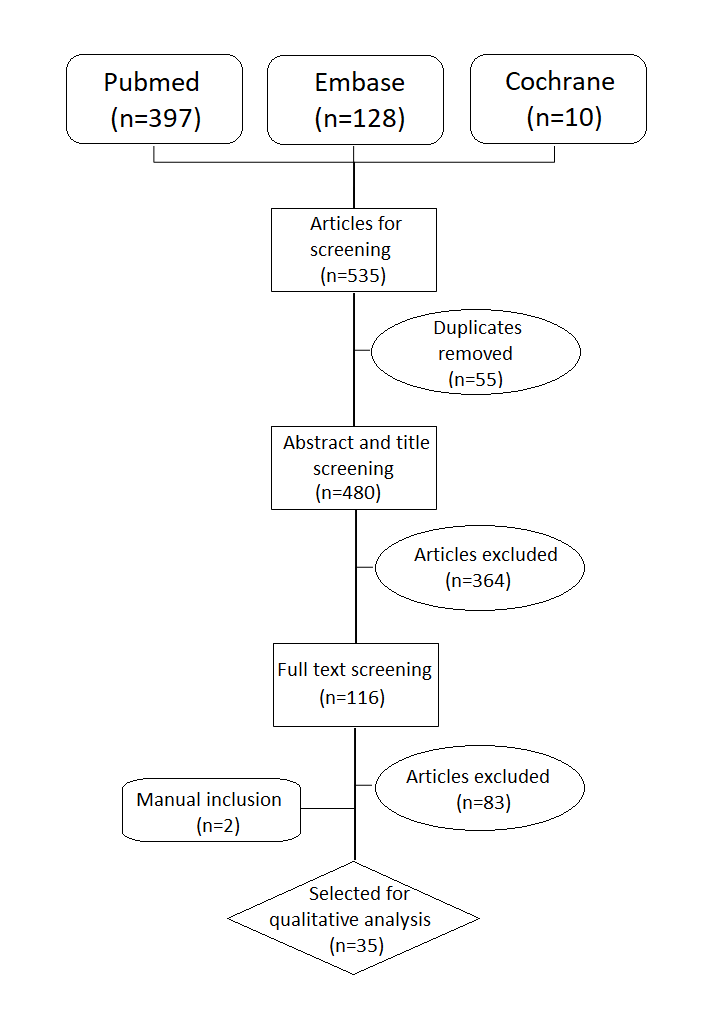
**

**Supplementary material 1. PRISMA 2009 Checklist**

| **Section/topic** | **#** | **Checklist item** | **Page** |
| --- | --- | --- | --- |
| **TITLE** | | |  |
| **Title** | **1** | **Identify the report as a systematic review, meta-analysis, or both.** | **1** |
| **ABSTRACT** | | |  |
| **Structured summary** | **2** | **Provide a structured summary including, as applicable: background; objectives; data sources; study eligibility criteria, participants, and interventions; study appraisal and synthesis methods; results; limitations; conclusions and implications of key findings; systematic review registration number.** | **2** |
| **INTRODUCTION** | | |  |
| **Rationale** | **3** | **Describe the rationale for the review in the context of what is already known.** | **3** |
| **Objectives** | **4** | **Provide an explicit statement of questions being addressed with reference to participants, interventions, comparisons, outcomes, and study design (PICOS).** | **4** |
| **METHODS** | | |  |
| **Protocol and registration** | **5** | **Indicate if a review protocol exists, if and where it can be accessed (e.g., Web address), and, if available, provide registration information including registration number.** |  |
| **Eligibility criteria** | **6** | **Specify study characteristics (e.g., PICOS, length of follow-up) and report characteristics (e.g., years considered, language, publication status) used as criteria for eligibility, giving rationale.** | **4-5** |
| **Information sources** | **7** | **Describe all information sources (e.g., databases with dates of coverage, contact with study authors to identify additional studies) in the search and date last searched.** | **5** |
| **Search** | **8** | **Present full electronic search strategy for at least one database, including any limits used, such that it could be repeated.** | **4** |
| **Study selection** | **9** | **State the process for selecting studies (i.e., screening, eligibility, included in systematic review, and, if applicable, included in the meta-analysis).** | **5** |
| **Data collection process** | **10** | **Describe method of data extraction from reports (e.g., piloted forms, independently, in duplicate) and any processes for obtaining and confirming data from investigators.** | **6** |
| **Data items** | **11** | **List and define all variables for which data were sought (e.g., PICOS, funding sources) and any assumptions and simplifications made.** | **4-6** |
| **Risk of bias in individual studies** | **12** | **Describe methods used for assessing risk of bias of individual studies (including specification of whether this was done at the study or outcome level), and how this information is to be used in any data synthesis.** | **5-6** |
| **Summary measures** | **13** | **State the principal summary measures (e.g., risk ratio, difference in means).** | **4-5** |
| **Synthesis of results** | **14** | **Describe the methods of handling data and combining results of studies, if done, including measures of consistency (e.g., I^2^) for each meta-analysis.** | **5-6** |

| **Section/topic** | **#** | **Checklist item** | **Page** |
| --- | --- | --- | --- |
| **Risk of bias across studies** | **15** | **Specify any assessment of risk of bias that may affect the cumulative evidence (e.g., publication bias, selective reporting within studies).** | **6** |
| **Additional analyses** | **16** | **Describe methods of additional analyses (e.g., sensitivity or subgroup analyses, meta-regression), if done, indicating which were pre-specified.** |  |
| **RESULTS** | | |  |
| **Study selection** | **17** | **Give numbers of studies screened, assessed for eligibility, and included in the review, with reasons for exclusions at each stage, ideally with a flow diagram.** | **6, figure 1, table 1** |
| **Study characteristics** | **18** | **For each study, present characteristics for which data were extracted (e.g., study size, PICOS, follow-up period) and provide the citations.** | **table 1** |
| **Risk of bias within studies** | **19** | **Present data on risk of bias of each study and, if available, any outcome level assessment (see item 12).** | **6, table 1** |
| **Results of individual studies** | **20** | **For all outcomes considered (benefits or harms), present, for each study: (a) simple summary data for each intervention group (b) effect estimates and confidence intervals, ideally with a forest plot.** | **table 1** |
| **Synthesis of results** | **21** | **Present results of each meta-analysis done, including confidence intervals and measures of consistency.** | **6-13, table 1** |
| **Risk of bias across studies** | **22** | **Present results of any assessment of risk of bias across studies (see Item 15).** | **table 1** |
| **Additional analysis** | **23** | **Give results of additional analyses, if done (e.g., sensitivity or subgroup analyses, meta-regression [see Item 16]).** |  |
| **DISCUSSION** | | |  |
| **Summary of evidence** | **24** | **Summarize the main findings including the strength of evidence for each main outcome; consider their relevance to key groups (e.g., healthcare providers, users, and policy makers).** | **13-15** |
| **Limitations** | **25** | **Discuss limitations at study and outcome level (e.g., risk of bias), and at review-level (e.g., incomplete retrieval of identified research, reporting bias).** | **15** |
| **Conclusions** | **26** | **Provide a general interpretation of the results in the context of other evidence, and implications for future research.** | **13-15** |
| **FUNDING** | | |  |
| **Funding** | **27** | **Describe sources of funding for the systematic review and other support (e.g., supply of data); role of funders for the systematic review.** | **15** |

***From:*  Moher D, Liberati A, Tetzlaff J, Altman DG, The PRISMA Group (2009). Preferred Reporting Items for Systematic Reviews and Meta-Analyses: The PRISMA Statement. PLoS Med 6(7): e1000097. doi:10.1371/journal.pmed1000097**

**Supplementary material 2. Search terms**

For PUBMED

(("Spondylarthropathies"[Mesh]) OR ("Spondylarthritis"[Mesh]) OR ("Spondylitis, Ankylosing"[Mesh]) OR (“Bechterew Disease”[Title/Abstract]) OR (“Bechterew's Disease”[Title/Abstract]) OR (“Ankylosing Spondyloarthritis”[Title/Abstract]) OR (“Ankylosing Spondylarthritis”[Title/Abstract]) OR (“Ankylosing Spondylitis”[Title/Abstract]) OR (“Spondylarthritis Ankylopoietica”[Title/Abstract]) OR (“Rheumatoid Spondylitis”[Title/Abstract]) OR (“Spondylitis Ankylopoietica”[Title/Abstract]) OR (“Ankylosing Spondyloarthritis”[Title/Abstract]) OR (Spondyloarthritid*[Title/Abstract]) OR (Spondyloarthrit*[Title/Abstract]) OR (Spondylarthritid*[Title/Abstract]) OR (“Spinal Arthritis”[Title/Abstract]))

AND

(("Heart Valves"[Mesh]) OR ("Heart Valve Diseases"[Mesh]) OR ("Heart Valve Prosthesis Implantation"[Mesh]) OR ("Cardiac Valve Annuloplasty"[Mesh]) OR ("Subaortic Bump"[Title/Abstract]) OR ("Subaortic Ridge"[Title/Abstract]) OR ("Pulmonary Stenosis"[Title/Abstract]) OR aorti*[Title/Abstract] OR mitral*[Title/Abstract] OR Regurg*[Title/Abstract] OR valv*[Title/Abstract] OR echocardiograph*[Title/Abstract])

NOT ("Clinical Conference"[Publication Type] OR "Congress"[Publication Type] OR "Consensus Development Conference"[Publication Type] OR "Editorial"[Publication Type] OR "Published Erratum"[Publication Type] OR "Letter"[Publication Type] OR "Comment"[Publication Type])

NOT ("Animals"[Mesh] NOT ("Animals"[Mesh] AND "Humans"[Mesh]))

FOR EMBASE

('spondyloarthropathy'/exp OR 'spondylarthritis'/exp OR 'ankylosing spondylitis'/exp OR

(“Bechterew Disease”:ab,ti) OR (“Ankylosing Spondyloarthritis”:ab,ti) OR

(“Ankylosing Spondylarthritis”:ab,ti) OR (“Ankylosing Spondylitis”:ab,ti) OR

(“Spondylarthritis Ankylopoietica”:ab,ti) OR (“Rheumatoid Spondylitis”:ab,ti) OR (“Spondylitis

Ankylopoietica”:ab,ti) OR (“Ankylosing Spondyloarthritis”:ab,ti) OR (Spondyloarthritides*:ab,ti)

OR (Spondyloarthrit*:ab,ti) OR (Spondylarthritid*:ab,ti) OR (“Spinal Arthritis”:ab,ti) OR

'spondylosis'/exp)

AND

(("Heart Valves"/exp) OR ("Heart Valve Diseases"/exp) OR ("Heart Valve Prosthesis

Implantation"/exp) OR ("Cardiac Valve Annuloplasty"/exp)

OR (“Valvular Heart Disease”:ab,ti) OR ("Aortic Valve Regurgitation":ab,ti) OR ("Aortic Valve

Incompetence":ab,ti) OR ("Aortic Stenosis":ab,ti) OR ("aortic valve stenosis":ab,ti) OR

("Subvalvular Aortic Stenosis":ab,ti) OR ("Subaortic Bump":ab,ti) OR ("Subaortic Ridge":ab,ti)

OR ("mitral valve regurgitation":ab,ti) OR ("mitral valve incompetence":ab,ti) OR ("mitral

regurgitation":ab,ti) OR ("mitral incompetence":ab,ti) OR ("mitral insufficiency":ab,ti) OR

("mitral valve stenosis":ab,ti) OR ("mitral valve regurgitation":ab,ti) OR ("mitral

regurgitation":ab,ti) OR ("mitral stenosis":ab,ti) OR ("Pulmonary Regurgitation":ab,ti) OR

("Pulmonary Valve Insufficiency":ab,ti) OR ("Pulmonary Valve Stenosis":ab,ti) OR ("Pulmonary

Stenosis":ab,ti) OR ("Cardiac Valve Annuloplasty":ab,ti) OR ("Heart Valve Replacement":ab,ti)

OR aorti*:ab,ti OR mitral*:ab,ti OR Regurg*:ab,ti OR valv*:ab,ti OR echocardiograph*:ab,ti)

AND ([embase]/lim NOT [medline]/lim) AND ([english]/lim OR [korean]/lim OR [french]/lim OR

[spanish]/lim) NOT ('animals'/exp NOT ('animals'/exp AND 'humans'/exp)) NOT ([conference

abstract]/lim OR [conference paper]/lim OR [conference review]/lim OR [erratum]/lim OR

[letter]/lim OR [note]/lim OR [short survey]/lim)

FOR COCHRANE

ID Search Hits

#1 MeSH descriptor: [Spondylarthropathies] explode all trees 834

#2 MeSH descriptor: [Spondylarthritis] explode all trees 888

#3 MeSH descriptor: [Spondylitis, Ankylosing] explode all trees 544

#4 ("Bechterew Disease" or "Bechterew's Disease" or "Ankylosing Spondyloarthritis" or

"Ankylosing Spondylarthritis" or "Ankylosing Spondylitis" or "Spondylarthritis Ankylopoietica" or

Spondylitis or "Spondylitis Ankylopoietica" or "Ankylosing Spondyloarthritis" or Spondyloarthritid* or

Spondyloarthrit* or Spondylarthritid* or "Spinal Arthritis"):ab,ti 1375

#5 #1 or #2 or #3 888

#6 MeSH descriptor: [Heart Valves] explode all trees 1048

#7 MeSH descriptor: [Heart Valve Diseases] explode all trees 1583

#8 MeSH descriptor: [Heart Valve Prosthesis Implantation] explode all trees 795

#9 MeSH descriptor: [Cardiac Valve Annuloplasty] explode all trees 50

#10 ("Subaortic Bump" or "Subaortic Ridge" or "Pulmonary Stenosis" or aorti* or mitral* or Regurg*

or valv* or echocardiograph*):ab,ti 18255

#11 #6 or #7 or #8 or #9 or #10 18564

#12 #5 and #11 2

**Supplementary material 3. Articles not available on library sources**

1. Badui E, Jimenez J, Robles E, Garcia Rubi DE, Mintz G. [The heart and ankylosing spondylitis]. Archivos del Instituto de Cardiologia de Mexico. 1985;55(1):69-74.

2. Cavailles J, Welti JJ, Bouvrain Y. [AORTIC INSUFFICIENCY OF ANKYLOSING SPONDYLARTHRITIS]. Archives des maladies du coeur et des vaisseaux. 1965;58:153-69.

3. Machado H, Befeler B, Morales AR, Vargas A, Aranda J. Aortic insufficiency in Reiter's syndrome. Southern medical journal. 1976;69(7):955-7.

4. Pop G, Romero-Ayala LC, Bialostozky D, Medrano GA, Martinez Lavin M. [Cardiac involvement in ankylosing spondylitis]. Archivos del Instituto de Cardiologia de Mexico. 1985;55(1):63-7.

5. Takkunen J, Vuopala U, Isomaki H. Cardiomyopathy in ankylosing spondylitis. I. Medical history and results of clinical examination in a series of 55 patients. Annals of clinical research. 1970;2(2):106-12.

6. Valkenborgh P, Dequeker J, Gielen F, H DEG. Arthritis and heart lesions. A study of 25 cases with pericarditis or valvular lesions associated to inflammatory joint disease. Acta cardiologica. 1976;31(4):269-76.

7. Good AE. Reiter's disease: a review with special attention to cardiovascular and neurologic sequellae. Seminars in arthritis and rheumatism. 1974;3(3):253-86.

8. Achuthan K, Porkodi R, Ramakrishnan S, Krishnamurthy V, Madhavan R, Parthiban M, et al. Pattern of rheumatic diseases in south India. V. Ankylosing spondylitis. A clinical and radiological study. The Journal of the Association of Physicians of India. 1990;38(10):774-6.

9. Ben Taarit C, Kaffel D, Ben Maiz H, Khedher A. [Cardiovascular manifestations in ankylosing spondylitis. Concerning 210 cases]. La Tunisie medicale. 2008;86(6):546-9.

10. Bontoux D, Bastin R, Coste F. [Ankylosing spondylarthritis (oculo-urethro-synovial syndrome?), aortic insufficiency and bacterial endocarditis]. Revue du rhumatisme et des maladies osteo-articulaires. 1967;34(10):592-6.

11. Castaneda S, Gonzalez-Juanatey C, Gonzalez-Gay MA. Inflammatory Arthritis and Heart Disease. Current pharmaceutical design. 2018.

12. Cavailles J. [Aortic insufficiency in ankylosing spondylitis]. Coeur et medecine interne. 1967;6(4):425-9.

13. Cosh JA. The heart and the rheumatic diseases. Rheumatology and physical medicine. 1972;11(6):267-80.

14. Cossio PR, Garcia Morteo O, Caruso AC. Ankylosing spondylitis and aortic insufficiency. Sistole. 1973;25(3):197-202.

15. Davidson P, Baggenstoss AH, Slocumb CH, Daugherty GW. CARDIAC AND AORTIC LESIONS IN RHEUMATOID SPONDYLITIS. Proceedings of the staff meetings Mayo Clinic. 1963;38:427-35.

16. Debray-Meignan S, Amor B, Delbarre F. Cardiac manifestations in ankylosing spondylitis. Rhumatologie - Revue International de Rhumatologie. 1979;8(5):345-56.

17. Dunn FG, Spencer DG, Bastian B. Clinical and echocardiographic assessment of the heart in ankylosing spondylitis. Rhumatologie - Revue International de Rhumatologie. 1980;10(3):167-72.

18. Jimenez-Balderas FJ, Martinez-Osuna P, Arellano J, Lara C, Yanez-Sanchez P, Camargo-Coronel A, et al. Does serum rheumatoid factor have an influence on the clinical picture of ankylosing spondylitis? Clinical and experimental rheumatology. 1997;15(3):289-93.

19. Kochbati S, Ben Miled M, Boussema F, Ketari S, Ben Maatallah Kochbati A, Kraiem S, et al. [Cardiac involvement in spondylarthropathies]. La Tunisie medicale. 2004;82(12):1082-90.

20. Lange U, Stapfer G, Ditting T, Geiger H, Teichmann J, Muller-Ladner U, et al. Pathologic alterations of the heart and the kidney in patients with ankylosing spondylitis. European journal of medical research. 2007;12(12):573-81.

21. Malette WG, Eiseman B, Danielson GK, Mazzoleni A, Rams JJ. Rheumatoid spondylitis and aortic insufficiency. An operable combination. The Journal of thoracic and cardiovascular surgery. 1969;57(4):471-4.

22. Marion J, Chagnon A, Le Gall F. Cardiac involvement in spondylarthritis ankylopoietica. MARSEILLE MED. 1973;110(7-8):569-75.

23. Nanda CN, Bose SL. ANKYLOSING SPONDYLITIS WITH AORTIC INSUFFICIENCY. Journal of the Indian Medical Association. 1964;43:550-1.

24. Paternotte L, Brochot P, Pennaforte JL, Eschard JP, Etienne JC. Aortic regurgitation in spondylarthropathies. Rhumatologie. 1991;43(3):57-65.

25. Rennke H, Losada M, Noguera H, Donoso S. Aortic incompetence of rheumatic origin. Rhumatologie - Revue International de Rhumatologie. 1973;3(3):257-62.

26. Roberts WC, Dangel JC, Bulkley BH. Nonrheumatic valvular cardiac disease: a clinicopathologic survey of 27 different conditions causing valvular dysfunction. Cardiovascular clinics. 1973;5(2):333-446.

27. Serre H, Simon L, Barjon MC, Lamboley C. [Obstructive arteritis of the aortic arch and the large aortic trunks and inflammatory rheumatism]. Revue du rhumatisme et des maladies osteo-articulaires. 1968;35(7):382-95.

28. Štolfa J, Kodeda M, Gollerová V, Štorková L, Galatíková D, Kopsa P, et al. Psoriatric arthritis. Ceska Revmatologie. 2002;10(2):76-81.

29. Titus JL. Rheumatic and collagen involvement of the heart. Cardiovascular clinics. 1972;4(2):307-23.

30. Vacrinos E. [The heart in chronic rheumatic diseases]. Revue du rhumatisme et des maladies osteo-articulaires. 1967;34(6):375-80.

**Supplementary material 4. Exclusion criteria for full-text reviewed articles**

| **Reference** | **Exclusion criteria** |
| --- | --- |
| Bergfeldt et al, 1997[1] | Wrong design |
| Boyer et al, 1999[2] | Insufficient data |
| Braun et al, 2017[3] | Insufficient data |
| Castaneda et al, 2017[4] | Insufficient data |
| Chan et al, 2006[5] | Insufficient data |
| Daien et al, 2019[6] | Insufficient data |
| Deesomchok et al, 1985[7] | Insufficient data |
| Gensler et al, 2015[8] | Wrong design |
| Gijon Banos et al, 1987[9] | Insufficient data |
| Gladman et al, 1998[10] | Wrong design |
| Good et al, 1974[11] | Wrong design |
| Heeneman et al, 2007 [12] | Wrong design |
| Hollingworth et al, 1979[13] | Wrong population |
| Johnsen et al, 2009[14] | Insufficient data |
| Kaarela et al, 1989[15] | Insufficient data |
| Kinsella et al, 1974[16] | Insufficient data |
| Kinsella et al, 1966[17] | Insufficient data |
| Kucuk et al, 2018[18] | Insufficient data |
| Lauterman et al, 2002[19] | Wrong design |
| Leirisalo-Repo et al, 1997[20] | Insufficient data |
| Lloyd et al, 1961[21] | Wrong design |
| Mader et al, 1999[22] | Insufficient data |
| Mielnik et al, 2018[23] | Insufficient data |
| Milaniuk et al, 2015[24] | Wrong design |
| Momeni et al, 2011[25] | Wrong design |
| Nagyhegyi et al, 1988[26] | Wrong design |
| Ninet et al, 1983[27] | Insufficient data |
| Nitter-Hauge et al, 1981[28] | Insufficient data |
| Olivieri et al, 1998[29] | Wrong design |
| Ozkan et al, 2016[30] | Wrong design |
| Palazzi et al, 2008[31] | Wrong design |
| Palazzi et al, 2011[32] | Wrong design |
| Perlroth et al, 1975[33] | Wrong design |
| Prakash et al, 1984[34] | Insufficient data |
| Roldan et al, 2008[35] | Wrong design |
| Saricaoglu et al, 2003[36] | Wrong population |
| Schilder et al, 1956[37] | Insufficient data |
| Schiotis et al, 2008[38] | Wrong design |
| Simpson et al, 1995[39] | Insufficient data |
| Slobodin et al, 2006[40] | Wrong design |
| Spangler et al, 1970[41] | Insufficient data |
| Spitzer et al, 1975[42] | Insufficient data |
| Tomas et al, 2013[43] | Wrong objective |
| Uusimaa et al, 2006[44] | Insufficient data |
| Van der Horst-Bruinsma et al, 2012[45] | Wrong design |
| Vazquez-Garcia et al, 1983[46] | Insufficient data |
| Vinsonneau et al, 2008[47] | Wrong design |
| Xu et al, 2014[48] | Insufficient data |
| Yates et al, 1975[49] | Insufficient data |
| Yuan et al, 2009[50] | Insufficient data |
| Zvaifler et al, 1963[51] | Wrong design |

1. Bergfeldt, L., HLA-B27-associated cardiac disease. Ann Intern Med, 1997. 127(8 Pt 1): p. 621-9.

2. Boyer, G.S., et al., Spondyloarthropathy in the community: clinical syndromes and disease manifestations in Alaskan Eskimo populations. J Rheumatol, 1999. 26(7): p. 1537-44.

3. Braun, J., et al., Cardiovascular Comorbidity in Inflammatory Rheumatological Conditions. Dtsch Arztebl Int, 2017. 114(12): p. 197-203.

4. Castaneda, S., C. Gonzalez-Juanatey, and M.A. Gonzalez-Gay, Sex and Cardiovascular Involvement in Inflammatory Joint Diseases. Clin Rev Allergy Immunol, 2017.

5. Chan, K.Y., et al., Effectiveness of a modified dosing regimen with a lower maintenance dose of infliximab in the treatment of patients with ankylosing spondylitis and spondyloarthropathy. APLAR Journal of Rheumatology, 2006. 9(2): p. 131-135.

6. Daien, C.I., et al., Relevance and feasibility of a systematic screening of multimorbidities in patients with chronic inflammatory rheumatic diseases. Joint Bone Spine, 2019. 86(1): p. 49-54.

7. Deesomchok, U. and T. Tumrasvin, Clinical study of Thai patients with ankylosing spondylitis. Clin Rheumatol, 1985. 4(1): p. 76-82.

8. Gensler, L.S., Axial spondyloarthritis: the heart of the matter. Clin Rheumatol, 2015. 34(6): p. 995-8.

9. Gijon Banos, J., et al., [Ankylosing spondylitis and cardiopathy]. Rev Clin Esp, 1987. 181(6): p. 323-6.

10. Gladman, D.D., Clinical aspects of the spondyloarthropathies. Am J Med Sci, 1998. 316(4): p. 234-8.

11. Good, A.E., Reiter's disease: a review with special attention to cardiovascular and neurologic sequellae. Semin Arthritis Rheum, 1974. 3(3): p. 253-86.

12. Heeneman, S. and M.J. Daemen, Cardiovascular risks in spondyloarthritides. Curr Opin Rheumatol, 2007. 19(4): p. 358-62.

13. Hollingworth, P., et al., Lone aortic regurgitation, sacroiliitis, and HLA B27. Case history and frequency of association. Br Heart J, 1979. 42(2): p. 229-30.

14. Johnsen, K., M. Mahonen, and P. Lunde, Prevalence estimation and follow-up of aortic regurgitation subjects in a Norwegian Sami population. Scand Cardiovasc J, 2009. 43(3): p. 176-80.

15. Kaarela, K., et al., Hidden psoriatic arthritis in seronegative oligoarthritis: a 14-year follow-up study. Clin Rheumatol, 1989. 8(4): p. 504-6.

16. Kinsella, T.D., L.G. Johnson, and R. Ian, Cardiovascular manifestations of ankylosing spondylitis. Can Med Assoc J, 1974. 111(12): p. 1309-11.

17. Kinsella, T.D., R.F. MacDonald, and L.G. Johnson, Ankylosing spondylitis: a late re-evaluation of 92 cases. Can Med Assoc J, 1966. 95(1): p. 1-9.

18. Kucuk, M., et al., Assessment of left atrial function using speckle tracking echocardiography in ankylosing spondylitis: a case-control study. Int J Cardiovasc Imaging, 2018. 34(12): p. 1863-1868.

19. Lautermann, D. and J. Braun, Ankylosing spondylitis--cardiac manifestations. Clin Exp Rheumatol, 2002. 20(6 Suppl 28): p. S11-5.

20. Leirisalo-Repo, M., et al., Long-term prognosis of reactive salmonella arthritis. Ann Rheum Dis, 1997. 56(9): p. 516-20.

21. Lloyd, O.C., Ankylosing Spondylitis with Mitral Stenosis: Clinico-Pathological Conference. Med J Southwest, 1961. 76(4): p. 132-142.

22. Mader, R., Atypical clinical presentation of ankylosing spondylitis. Semin Arthritis Rheum, 1999. 29(3): p. 191-6.

23. Mielnik, P., A.M. Hjelle, and J.L. Nordeide, Coexistence of Takayasu's arteritis and ankylosing spondylitis may not be accidental - Is there a need for a new subgroup in the spondyloarthritis family? Mod Rheumatol, 2018. 28(2): p. 313-318.

24. Milaniuk, S., et al., Influence of psoriasis on circulatory system function assessed in echocardiography. Arch Dermatol Res, 2015. 307(10): p. 855-61.

25. Momeni, M., N. Taylor, and M. Tehrani, Cardiopulmonary manifestations of ankylosing spondylitis. International Journal of Rheumatology, 2011. 2011.

26. Nagyhegyi, G., et al., Cardiac and cardiopulmonary disorders in patients with ankylosing spondylitis and rheumatoid arthritis. Clin Exp Rheumatol, 1988. 6(1): p. 17-26.

27. Ninet, J., et al., [Aortic insufficiencies in ankylosing spondylarthritis. Clinical study and prognosis of 12 cases]. Rev Med Interne, 1983. 4(1): p. 94-104.

28. Nitter-Hauge, S. and J.E. Otterstad, Characteristics of atrioventricular conduction disturbances in ankylosing spondylitis (Mb. Bechterew). Acta Med Scand, 1981. 210(3): p. 197-200.

29. Olivieri, I., et al., Clinical manifestations of seronegative spondylarthropathies. Eur J Radiol, 1998. 27 Suppl 1: p. S3-6.

30. Ozkan, Y., Cardiac Involvement in Ankylosing Spondylitis. J Clin Med Res, 2016. 8(6): p. 427-30.

31. Palazzi, C., et al., Aortic involvement in ankylosing spondylitis. Clin Exp Rheumatol, 2008. 26(3 Suppl 49): p. S131-4.

32. Palazzi, C., et al., Aortitis and periaortitis in ankylosing spondylitis. Joint Bone Spine, 2011. 78(5): p. 451-5.

33. Perlroth, M.G., Connective tissue diseases and the heart. Jama, 1975. 231(4): p. 410-2.

34. Prakash, S., et al., Ankylosing spondylitis in North India: a clinical and immunogenetic study. Ann Rheum Dis, 1984. 43(3): p. 381-5.

35. Roldan, C.A., Valvular and coronary heart disease in systemic inflammatory diseases: Systemic Disorders in heart disease. Heart, 2008. 94(8): p. 1089-101.

36. Saricaoglu, H., et al., Echocardiographic findings in subjects with psoriatic arthropathy. J Eur Acad Dermatol Venereol, 2003. 17(4): p. 414-7.

37. Schilder, D.P., W.P. Harvey, and C.A. Hufnagel, Rheumatoid spondylitis and aortic insufficiency. N Engl J Med, 1956. 255(1): p. 11-7.

38. Schiotis, R.E., et al., Ankylosing spondylitis and other spondyloarthropathies: Extra-articular manifestations and their management. Future Rheumatology, 2008. 3(5): p. 457-473.

39. Simpson, J., M.S. Borzy, and G.M. Silberbach, Aortic regurgitation at diagnosis of HLA-B27 associated spondyloarthropathy. J Rheumatol, 1995. 22(2): p. 332-4.

40. Slobodin, G., et al., Aortic involvement in rheumatic diseases. Clin Exp Rheumatol, 2006. 24(2 Suppl 41): p. S41-7.

41. Spangler, R.D., B.D. McCallister, and D.C. McGoon, Aortic valve replacement in patients with severe aortic valve incompetence associated with rheumatoid spondylitis. Am J Cardiol, 1970. 26(2): p. 130-4.

42. Spitzer, S., F. Peguero, and D. Mason, Rheumatoid spondylitis, aortic insufficiency, and coronary artery disease: an operable combination. Chest, 1975. 68(6): p. 828-9.

43. Tomas, L., et al., Acute and long-term effect of infliximab on humoral and echocardiographic parameters in patients with chronic inflammatory diseases. Clin Rheumatol, 2013. 32(1): p. 61-6.

44. Uusimaa, P., et al., Aortic valve insufficiency in patients with chronic rheumatic diseases. Clin Rheumatol, 2006. 25(3): p. 309-13.

45. van der Horst-Bruinsma, I.E. and M.T. Nurmohamed, Management and evaluation of extra-articular manifestations in spondyloarthritis. Therapeutic Advances in Musculoskeletal Disease, 2012. 4(6): p. 413-422.

46. Vazquez Garcia, R., et al., [Cardiovascular manifestations in ankylosing spondylitis]. Rev Esp Cardiol, 1983. 36(3): p. 205-12.

47. Vinsonneau, U., et al., Cardiovascular disease in patients with spondyloarthropathies. Joint Bone Spine, 2008. 75(1): p. 18-21.

48. Xu, L., J. Heath, and A. Burke, Ascending aortitis: a clinicopathological study of 21 cases in a series of 300 aortic repairs. Pathology, 2014. 46(4): p. 296-305.

49. Yates, D.B. and J.T. Scott, Cardiac valvular disease in chronic inflammatory disorders of connective tissue. Factors influencing survival after surgery. Ann Rheum Dis, 1975. 34(4): p. 321-5.

50. Yuan, S.M., Cardiovascular involvement of ankylosing spondylitis: report of three cases. Vascular, 2009. 17(6): p. 342-54.

51. Zvaifler, N.J. and A.M. Weintraub, Aortitis and aortic insufficiency in the chronic rheumatic disorders—A reappraisal. Arthritis & Rheumatism, 1963. 6(3): p. 241-245.
